# Scenario-Based Programming of Voice-Controlled Medical Robotic Systems

**DOI:** 10.3390/s22239520

**Published:** 2022-12-06

**Authors:** Adam Rogowski

**Affiliations:** Faculty of Mechanical and Industrial Engineering, Warsaw University of Technology, ul. Narbutta 86, 02-524 Warsaw, Poland; adam.rogowski@pw.edu.pl

**Keywords:** medical robots, man-machine communication, speech recognition

## Abstract

An important issue in medical robotics is communication between physicians and robots. Speech-based communication is of particular advantage in robot-assisted surgery. It frees the surgeon’s hands; hence, he can focus on the principal tasks. Man-machine voice communication is the subject of research in various domains (industry, social robotics), but medical robots are very specific. They must precisely synchronize their activities with operators. Voice commands must be possibly short. They must be executed without significant delays. An important factor is the use of a vision system that provides visual information in direct synchronization with surgeon actions. Its functions could be also controlled using speech. The aim of the research presented in this paper was to develop a method facilitating creation of voice-controlled medical robotic systems, fulfilling the mentioned requirements and taking into account possible scenarios of man-machine collaboration in such systems. A robot skill description (RSD) format was proposed in order to facilitate programming of voice control applications. A sample application was developed, and experiments were conducted in order to draw conclusions regarding the usefulness of speech-based interfaces in medical robotics. The results show that a reasonable selection of system functions controlled by voice may lead to significant improvement of man-machine collaboration.

## 1. Introduction

Within a relatively short period of time, robotic systems have been broadly introduced in many areas of medicine. For example, they may assist sonographers in performing ultrasound examinations by addressing common limitations of sonography: the physical fatigue and the difficulty of interpreting ultrasound data [1]. Robot-assisted stereotactic laser ablation (SLA) could offer an accurate, efficient, minimally invasive, and safe method for placement of an ablation catheter into the target [2]. Broadly understood medical robotics also comprises the domain of robotic prostheses [3]. Another domain of robot applications, partially related to medicine, is healthcare robotics, which are rapidly developing [4]. The most advanced development, however, has probably been achieved in robot-assisted surgery.

Generally, there are two main subcategories of robotic surgical assistants: surgeon extender robots and auxiliary surgical support robots. Surgeon extender robots manipulate surgical instruments under direct control of the surgeon. Their primary advantage consists in overcoming the perception and manipulation limitations of the surgeon. Auxiliary surgical support robots generally work alongside the surgeon and perform such routine tasks as tissue retraction, limb positioning, or endoscope holding. Their main advantages are: reduction in the number of people required in the operating room, improved task performance (e.g., a steadier endoscopic view), and safety (e.g., elimination of excessive retraction forces) [5].

The use of surgical robots is of particular advantage in Minimally Invasive Surgery (MIS). For example, the applicability of remote-controlled robotic surgery to laparoscopic radical prostatectomy was already proven by successful operation 20 years ago [6]. Raytis et al. claim that, after completing over 250 procedures with the da Vinci Xi surgical system and noting the anesthetic implications, the conclusion may be drawn that the use of this system may lead to decreases in operating times and to increased patient safety [7].

Aside from MIS, there are also concepts of introducing Natural Orifice Transluminal Endoscopic Surgery (NOTES) [8]. For example, some preliminary research was conducted with the aim of designing miniaturized robots able to completely enter the body and to perform surgical procedures under intuitive teleoperation [9]. The NOTES concept also stood behind experiments with continuum robot manipulators intended for application in interventional medicine [10].

An important issue in medical robotics is communication between physician and robot. Speech-based communication would be of particular advantage in robot-assisted surgery because it frees the hands of surgeon and lets him focus on the principal tasks. As stated by Matarneh et al., application of voice communication based on natural language should allow quicker control of the medical robot and provide increased accuracy in intercranial operations. Therefore, they proposed the structure of voice command language for this domain. However, the work seems to have a purely theoretical character, as they provide no information on any experiments [11].

Generally, the literature referring to man-robot speech-based communication is quite rich, although the majority of scientific papers devoted to this subject are not directly related to medical applications. Nevertheless, some of the presented methods and solutions could also be applied in medical robots if the specific requirements and conditions related to medical robotics were taken into account.

A part of the reported research is devoted to industrial applications [12]. Proposed solutions are related to various levels of man-machine interaction. Li et al. present applications of voice control in flexible manufacturing cells where very simple commands are used to operate individual devices, e.g., clamps [13]. Bingol et al. deal with task-oriented voice communication with an industrial robot using the Turkish language [14]. In order to increase the effectiveness of speech-based communication, some authors present research combining voice control with other means of communication. For example, Shaikh et al. apply vision system for object tracking in order to recognize gestures [15]. Majewski and Kacalak use augmented reality in a speech-based interface for controlling mobile cranes [16].

Combining speech-based robot control with other means of communication is very popular in the domain of social robotics [17]. In a complex environment, it is not easy or even not possible to verbally express the task for a robot using short, simple commands. Long, complicated commands decrease the effectiveness of speech-based dialog and may lead to low recognition rates. In order to avoid the ambiguity of compact, concise commands, the voice communication may be supplemented by gestures, and many authors deal with various aspects of this topic [18,19,20,21]. There are also works dealing with the combination of voice and haptic control [22] or voice and operator’s gaze monitoring [23].

Some research has also been performed in order to provide context for voice commands using automatic recognition of objects in a robot’s environment. This can be achieved using laser scanners and sonars, [24] as well as analysis of object images derived from the vision system [25,26].

As far as speech-based man-robot communication in the medical domain is concerned, the amount of scientific papers devoted to this topic is not very big. Some of them deal with aspects that are only partially related to medicine. For example, Pramanik et al. deal with the design of voice-controlled robots which can work round the clock as assistants in hospitals [27]. They developed a simple system based on Arduino and Raspberry Pi. Depending on the voice command uttered, the mobile robot grasps and brings an object of a given color (the system is equipped with automatic image recognition software). An application only partially related to medicine has been developed by Grasse et al. In connection with the COVID-19 pandemic, the subject of their research was a speech-controlled, self-sanitizing robot that enabled safe delivery of items to residents of a care facility [28]. Because hands-free interaction was required, natural language had to be used. The speech-based interface had to collaborate with the vision system used for mapping, navigation, and obstacle avoidance. The authors also tested the effects of face masks on speech recognition interface functioning. Therefore, the results of their research could possibly also be used in robot-assisted surgery, where the robot operator (surgeon) may also wear a mask.

Research more directly related to medical robots was presented by Gundogdu et al., who dealt with the development of a voice control system for a prosthetic robot arm [29].

As far as surgical robots are concerned, a study on speech-based control has been performed by Zinchenko et al. They dealt with control of an assistant robot helping the surgeon by handling the endoscope [30]. Similarly, Vaida et al. presented a voice-controlled parallel robot for minimally invasive surgery, used for laparoscope camera positioning [31]. Simple voice commands were used to position the camera. Microsoft Speech SDK was used to develop the voice control module.

As can be concluded from foregone studies, man-machine voice communication in the area of medical robotics, quite particularly in surgical applications, is very specific. This results from the manner in which the robots are used. In contrast to social or industrial robots, which are usually controlled by task-oriented voice commands and should show a high level of autonomy, medical robots must precisely synchronize their activity with the operators. The voice commands must be possibly short, and their execution must be performed without significant delay. Another important factor is the use of a vision system, which should provide visual information in direct synchronization with surgeon actions. Once speech-based communication is introduced, it can be applied not only to the control of the robot itself, but also create quite new possibilities with regard to vision systems. Generally, an endoscopic camera is used to provide the surgeon with the current view of the area of interest. The surgeon (or his assistant) may navigate the endoscope in order to change the viewpoint, but any advanced operations on the image are usually performed later, using appropriate computer programs. Contemporary image processing systems offer a very rich spectrum of possible options: from simple zooming on the area of interest to such advanced techniques as instrument tracking, suspicious lesion tracking, or endoscopic view expansion based on 3D surface reconstruction [32]. According to Boskoski and Costamagna [33], the future applications and developments of endoscopy robots will include high-resolution imaging, 3D vision, and advanced optical analysis.

Medical vision systems are often based on artificial intelligence. For example, deep learning techniques have been used in several areas of endoscopy, including polyp detection and classification, diagnosis of infections, detection of cancer, etc. [34]. Medical applications of artificial intelligence are the subject of research performed by numerous scientific teams. Wimmer et al. deal with improvement of computer-assisted endoscopic image diagnosis [35]. They retrieved additional teaching data for a convolutional neural network (CNN) from endoscopic videos under different image recording conditions (different viewpoints and image qualities). Ali et al. developed a method using CNN for restoration of corrupted video frames for the purpose of facilitating automated analysis of endoscopy videos [36]. Zheng et al. developed a two-stage training method (pre-training process and implicit regularization training process) to reduce the overfitting problem in CNN [37]. Although their solution is not directly focused on medical applications, it could be possibly applied also in this domain. Similarly, the work by Jin et al., dealing with an interesting problem of generating depth maps for 2D face images, could also be of interest in medical imaging [38].

Various aspects of cell segmentation and tracking are the subject of intensive research [39,40]. Their results can be used in biomedical image analyses, such as quantifying cells, cell nuclei, and other submillimeter structures from microscope images [40].

The use of advanced options provided by image processing systems during the operation is, however, restricted by the fact that the surgeon has his hands engaged with endoscope navigation anyway. Voice control opens quite new prospects here because the voice commands can be addressed at the robotic assistant as well as at the image analysis system.

The research presented in this paper started with analysis of possible scenarios of man-machine collaboration. Its final aim was to develop a method facilitating creation of voice-controlled medical robotic systems, fulfilling the previously mentioned requirements. Based on this method, a sample system for voice control was developed. Experiments conducted using that system allowed us to draw conclusions regarding the functioning of speech-based interfaces in medical robotics.

## 2. Materials and Methods

The method presented in this paper is based on the following assumptions regarding man-machine speech communication:

- The user may communicate by voice with the robotic assistant, as well as with the graphical user interface (GUI) of the system providing visual information derived from the camera;
- Voice commands addressed to the robot will refer to the parameters of its motion: direction, distance, and speed;
- Voice commands addressed to the GUI of the vision system should make it possible to use its main functionalities in a simple way, e.g., focusing on objects of interest shown on the image, zooming in and out, image panning and rotation, etc.

### 2.1. General Conception of Speech-Based Man-Robot Communication

In order to facilitate programming of voice control applications based on the assumptions listed above, the solution presented in this paper consists in the one-time development of a system “core” by a professional programmer, whereas adjustment to particular applications should be performed easily and swiftly in a transparent way.

The first step of this research was to analyze what types of man-machine speech-based communication scenarios are typical for medical robotics. Three main types of scenarios were distinguished:

- Scenarios based on simple, sequential voice commands;
- Scenarios based on multiple choices;
- Scenarios based on continuous dialog.

Scenarios based on simple, sequential commands assume that each command is followed by an action determined by this command, and the next command cannot be uttered until this action is complete. A sample scenario could look like this:

- Command: *Robot, move the tool ten millimeters to the left;*
- Action: Robot moves left;
- Command: *Robot, move the tool twenty millimeters up;*
- Action: Robot moves up.
- …

Commands used in such scenarios must fully describe the action of the robot, including all necessary parameters (in the above example: distance in millimeters). This may sometimes result in quite long and complex commands and make the voice dialog less effective. Therefore, such scenarios should be performed in relatively uncomplicated situations. On the other hand, programming of these scenarios is rather simple; therefore, the majority of voice-control systems, particularly industrially oriented ones, operate in this way.

It is worth mentioning that this art of medical robot control (where the motion length values are determined) is directly associated with the introduction of speech communication. Let us present it on the example of endoscope handling. Generally, contemporary practice in endoscopy consists mainly in manual operation; thus, the surgeon makes a decision on displacement length according to what he can see on the screen and prescinds from determination of this length in length units. As stated by Zinchenko et al., in the verbal communication between surgeon and his human assistant, it is common to use ambiguous commands, such as “a little to the right” or “move more to the left”. The assistant must understand how far the endoscope should be moved to achieve the desired view [30]. Introduction of robotic endoscope holders alone does not change the situation in this regard: the surgeon operates a foot pedal in order to move the endoscope, but the length of motion still depends on what he currently can see on the screen. However, introduction of verbal communication with the robotic assistant may lead to situations where motion commands based on displacement value are needed. Of course, a question must be asked about the units in which the displacement is given. Usually, endoscope translational motions are restricted to its introduction and retraction (this would correspond to voice commands like “forwards” or “back”), whereas moving to the sides (which would correspond to voice commands like “left”, “right”, “up”, and “down”) is practically performed by rotating the flexible end of the endoscope. Therefore, it is questionable what the displacement units should be, e.g., millimeters, degrees, or references to dimensions of objects on the screen (e.g., “half of polyp’s length”). In any case, appropriate calibration will be necessary. Due to the lack of past experience in this area, the question is still open. The method proposed in this paper lets the voice commands be defined freely. In the application presented in the next section, commands such as “move five millimeters to the right” are used. It seems that in some situations, they could be useful for a surgeon who has good knowledge of the range of dimensions of the objects depicted on the screen (granted that appropriate calibration was previously performed in order to determine what endoscope motion corresponds to the motion parameters determined in the voice command).

Scenarios based on multiple choices are performed in certain situations when the required robot action cannot be described in a simple way because it involves complex objects shown on the screen, e.g.:

- Command: *Enlarge the blood cell;*
- Action: A blood cell is recognized and the appropriate part of the image is enlarged;
- Command: *The next one;*
- Action: Another blood cell is searched and the appropriate part of the image is enlarged;
- Command: *The previous one;*
- Action: The previously enlarged part of the image is shown.
- …

Due to the ambiguity of the first command, such scenarios are performed in a loop which may comprise recognition of multiple auxiliary voice commands, resulting in automatic search for new instances of the object described in the first command. The voice dialog may have a complex structure and a flexible length. It can be interrupted by a voice command associated with another scenario. This means that the speech recognition engine must simultaneously listen for possible auxiliary commands, as well as for commands associated with other scenarios. This results in a more complicated algorithm responsible for the activation of sublanguage currently used by the speech recognition engine. On the other hand, the auxiliary commands can be quite simple because they refer to information contained in the first command. This increases the effectiveness of voice dialog.

It is worth mentioning that this way of operation is purposeful only in situations where a restricted number of objects are shown on the screen. In other situations, we can either move the mouse cursor to the appropriate point (a simple and swift action, but it engages the hands) or apply gaze detection to select a particular image area (it does not engage the hands, but requires installing an additional system).

The use of medical robots, in particular in robot-assisted surgery, may result in situations when scenarios based on continuous dialog have to be performed. For example, when the task of a robot is to hold an endoscope during the surgery performed by a doctor, the robot operator (surgeon) often cannot determine in advance all details of the action to be performed by the robot, e.g., how long the robot is expected to move in a given direction, because those details depend on changing circumstances. A good example is the surgeon command “*Move endoscope left*”. In this case, for example, the termination of the motion depends on what the doctor will detect on the screen and when.

Various solutions to this problem can be found. Zinchenko et al. [30] propose that while the operator is uttering the voice command, the speech recognition machine continuously recognizes the currently pronounced phonemes in order to adjust the length of the robot motion to the time taken to utter the whole command. For example, the robot motion will be longer for a long command of “l-eh-eh-ft” than for a short command of “left”. Such a solution lets the verbal dialog between man and machine be very effective because it is performed in a very natural way. There are also, however, two disadvantages:

- The voice recognition system cannot be created on any standard platform because it must interfere with the speech recognition engine in real-time on the phoneme level, and this is not always possible;
- Application of such a method is limited to short motions only, due to limitations regarding uttering a single vowel by humans.

The solution proposed in this paper assumes that the voice dialog is based on complex commands, consisting of the main phrase activating the robot motion, the termination phrase, and optional modification phrases (responsible for changes in motion parameters, e.g., speed). A sample scenario could look like this:

- Main phrase: *Move endoscope left;*
- Action: Endoscope starts to move in appropriate direction;
- Modification phrase: *Faster;*
- Action: Velocity is increased;
- Modification phrase: *Slow down;*
- Action: Velocity is decreased;
- …
- Termination phrase: *Stop;*
- Action: Endoscope stops.

### 2.2. Scenario-Based Programming of Robotic Systems

As mentioned before, the solution presented in this paper assumes development of a program “core” comprising algorithms performing scenario types described in the previous section, including appropriate activation and deactivation of groups of voice commands belonging to the language defined for a given application. The “core” will be developed once only. In this way, in order to create a new application, it will be enough to describe the command language and to specify details of scenarios.

As far as voice command language description is concerned, VCD format will be used [41,42]. This format is very convenient because it lets us define both syntax and semantics of voice commands in a very easy and transparent way. In order to explain its structure, a sample command description in VCD will be now presented. Let us assume the command refers to robot linear motion in a given direction with a given distance. First, the phrases describing direction, distance value, and distance unit have to be defined:

*#def direction*

*left: −1*

*right: 1*

*#def value*

*…*

*five: 5*

*six: 6*

*…*

*#def unit*

*millimeters: 1*

*centimeters: 0.1*

*decimeters: 0.01*

A sample voice command description based on these phrases would look like this:

*#com move*

move *value *units *direction ?please: p2*p3*p4

The phrase description comprises all possible variants of the phrase formulation. In order to facilitate semantic analysis, one or more numerical parameters may be assigned to each variant. Description of both commands and phrases may contain optional words or phrases (preceded by the “?” sign).

Semantic analysis of a voice command consists in the calculation of command parameters using the values of parameters describing its component phrases. Variables p1, p2, p3, p4… denote the values assigned to the first, second, third, fourth… phrase/word of the voice command. In the above example, the first word (“move”) is an indispensable part of this command, but it does not carry any numerical values needed to perform semantic analysis. In contrast, the numerical values carried by the second, third, and fourth word (p2, p3, p4) determine the length of displacement; hence, the numerical parameter of the command move as a whole is calculated as product of p2, p3, and p4. The resulting value equals the required displacement in millimeters (independently of what units were used by the operator uttering the command). Motion to the right is determined by positive value, whereas motion to the left is determined by negative value. The value of the command parameter may then be sent to the robotic system control. More details can be found in [41].

In this research, it is proposed that details of scenarios are determined using description of robot “skills” and their assignment to particular voice commands described using VCD format. The term “skill” is broadly used in the domain of autonomous robots. Industrial applications of so-called collaborative robots (cobots), communicating with their operators by voice, are often skill-based [43]. Although this is a different domain of robotics, the concept of industrial cobots is very similar to that of medical robots because it is based on direct human-robot collaboration. Wiese et al., who developed a flexible control for robot cells in manufacturing, state that skill-based control architecture lets us fulfill four basic requirements regarding effective robot control: extensibility, flexible usability, configurability, and reusability [44]. Another example of a skill-based approach is the work of Pane et al., who developed a programming framework that allows several skills to run synchronously in the presence of disturbances [45].

In the solution presented in this paper, the skills are executed separately, although the higher-level skills may execute the low-level skills. As the sequence in which particular skills are executed depends solely on voice commands uttered by the operator, the higher-level task programming (usually an important part of skill-based systems) is not present in this solution. The skills may refer to the robot, but also to other elements of the robotic system (e.g., GUI of the vision system).

Three types of skills, corresponding to the three types of scenarios discussed in the previous section, are proposed:

- Simple skills;
- Multiple choice skills;
- Continuous action skills.

Additionally, there are “auxiliary” skills corresponding to actions that should be undertaken as a response to auxiliary phrases uttered during the execution of multiple choice skills and continuous action skills.

In order to provide a uniform description of both voice commands and robot skills, a special format for robot skill description (RSD) was developed as a part of the research presented in this paper. The simple skills are described in the following form:

*#skill <name of skill>*

*type: simple*

*execute: <procedure call>*

*confirm: <procedure call>*

As the skills have to be unambiguously assigned to voice commands, their names must be equal to command names. In this way, command description in VCD will be associated with skill description in RSD.

The statement “execute” determines the application-specific procedure which is responsible for sending an appropriate execution code to the robot controller. This procedure call may also include numerical parameters p1, p2, p3…, which are derived from semantic analysis of voice commands (as described previously).

The statement “confirm” (which is optional) determines the application-specific procedure which is called when the skill execution is completed. For example, it may be a sentence uttered through the loudspeakers.

An example of voice command description in VCD and the corresponding description of a simple skill in RSD is presented below:

*#com move distance right*

move *distance *units to the right: p2*p3

*#skill move distance right*

*type: simple*

*execute: move right (p1)*

*confirm: speaker (end of motion)*

In the above example, the distance to be covered is calculated as the product of the values returned by the second and third phrase of the command (due to the lack of space, description of these phrases is not shown). This value is the first (and the only) numerical parameter of command. It is then passed to the application-specific procedure “move right” (it is referred to as “p1” in the procedure call). The above described skill “move distance right” is executed by operator’s command, e.g., “move five millimeters to the right”. After execution of the skill is completed, the confirmation “End of motion” is automatically uttered through the loudspeaker.

The multiple choice skills are described in the following form:

*#skill <name of skill>*

*type: choice*

*execute: <procedure call>*

*modify: <name of skill>, <name of skill>,…*

*confirm: <procedure call>*

As can be seen, in comparison with simple skills, the multiple choice skills may contain the additional statement “modify”. This statement contains a list of available options in the form of auxiliary skills which can be activated by voice in the process of multiple choice skill execution. The name of this statement (“modify”) results from the fact that those auxiliary skills may influence the way the skill is performed. An example of multiple choice skill description, together with auxiliary skills and corresponding voice commands, is presented below:

*#com enlarge*

*enlarge the blood cell*

*#com another*

the one *there: p3

*#def there*

*to the right: 1*

*to the left: 2*

*above: 3*

*below: 4*

*#skill enlarge*

*type: choice*

*execute: enlarge (0)*

*modify: another*

*#skill another*

*type: auxiliary*

*execute: enlarge (p1)*

In the above example, the main skill (“enlarge”) is executed as a result of the operator’s command “Enlarge the blood cell”, addressed at the vision system. Then the application-specific procedure “enlarge” is called with numerical parameter 0 (which means that the vision system focuses on any blood cell in the field of view). In order to provide the necessary data for potential future execution of the auxiliary skill, the coordinates of that blood cell must be stored in memory.

In contrast to simple skills, the system listens not only for other main commands, but also for all commands assigned to the skills listed in the “modify” statement of the currently executed skill. When one of those auxiliary commands is uttered by the operator (e.g., “The one to the left”), the auxiliary skill (“another”) is executed. As a result, the procedure “enlarge” is called AND ITS numerical parameter (p1) IS derived from semantic analysis of the auxiliary command.. Depending on the sentence uttered by the operator, and on the previously stored coordinate values of the blood cell that currently has the focus, the system determines the new part of the image that should be enlarged. The statement “confirm” is omitted in this example because enlargement of the appropriate part of the image is a sufficient confirmation of command execution.

The continuous action skills are described in the following form:

*#skill <name of skill>*

*type: continuous*

*execute: <procedure call>*

*modify: <name of skill>, <name of skill>,…*

*terminate: <name of skill>*

*confirm: <procedure call>*

The main difference between continuous action skills and the previously discussed types of skills consists in the manner in which the “execute” procedure is called. In contrast to other skill types, this procedure is not called only once, and its calls are performed in a loop. In this way, the action of the robot (or of another element of the robotic system) is performed continuously. The loop can be broken only when an appropriate voice command is uttered. Therefore, description of a continuous action skill must contain the statement “terminate”, determining the auxiliary skill (and the auxiliary command assigned to it) responsible for termination of the currently running continuous action.

An example of continuous action skill description, together with auxiliary skills (including termination skill) and corresponding voice commands, is presented below:

*#com move right*

*move right*

*#com slow down*

*slow down*

*attention*

*#com speed up*

*speed up*

*faster*

*#com stop*

*stop*

*terminate motion*

*#skill move right*

*type: continuous*

*execute: step right*

*modify: slow down, speed up*

*terminate: stop*

*#skill slow down*

*type: auxiliary*

*execute: decrease speed*

*#skill speed up*

*type: auxiliary*

*execute: increase speed*

*#skill stop*

*type: auxiliary*

*execute: stop motion*

The main loop of the continuous motion skill is started when the operator utters the command “move right”. Each pass of the loop executes the application-specific procedure “step right”, which causes a small robot motion with a pre-determined (default) speed. Continuous listening for voice commands is active all the time in this loop. When the operator activates any of the auxiliary “modify” skills by uttering the appropriate command (e.g., “faster”), the current speed value is automatically changed. The new value is then used by the “step right” procedure in all consecutive loop passes. When the user utters the sentence “stop” or “terminate motion”, the skill “stop” is executed and the loop is terminated. In contrast to skills of the type “choice”, listening for the auxiliary commands is then terminated immediately (not when a new main command is uttered). It is worth mentioning that during continuous action skill execution, there is no need to listen for commands other than those assigned to “modify” and “terminate” skills. Therefore, the possibility of wrong recognition is definitely decreased.

As far as auxiliary skills are concerned, the structure of their description in RSD is the same as that of simple skills (except the keyword “auxiliary” instead of “simple”). The main difference consists, however, in the way their corresponding commands are listened for. The commands assigned to simple skills are listened for all the time except for when the continuous action skills are executed, whereas the commands assigned to auxiliary skills are listened for only when multiple choice or continuous action skills are executed.

However, all the issues related to voice command listening are solved in the system core; hence, there is no need to deal with them when developing a particular application.

In order to demonstrate that the use of the method presented in this paper substantially increases the programming efficiency, the program code of a sample speech-based interface is presented in Supplementary Data S1 (file research data S1.doc). Its components are: voice command language description in VCD format (99 lines), skill description in RSD format (86 lines), and the execution module containing procedures for direct control of the robot (111 lines). Hence, creation of the whole application needs 296 lines of code. Previously written applications in the Pascal language, performing a similar task (referring to the same voice commands, as well as to the same procedures contained in the execution module), required about 3000 lines of code, i.e., ten times more. Although it is only an intuitive comparison, it is nevertheless obvious that the difference in programming efficiency with and without the use of the proposed method is huge.

## 3. Results

In order to test how the speech-based interface would function in practice, a system based on the method presented in this paper was developed. As far as the hardware is concerned, it comprises a five-axis robot and a camera connected to a custom image recognition system developed in our laboratory. The robot was a Mitsubishi Movemaster RV-M1 with a payload of 1.2 kg. External control of the robot was possible through a RS-232C serial interface (similar to experiments performed by Zinchenko et al. [30] with a HIWIN robotic endoscope holder).

**Figure 1:**
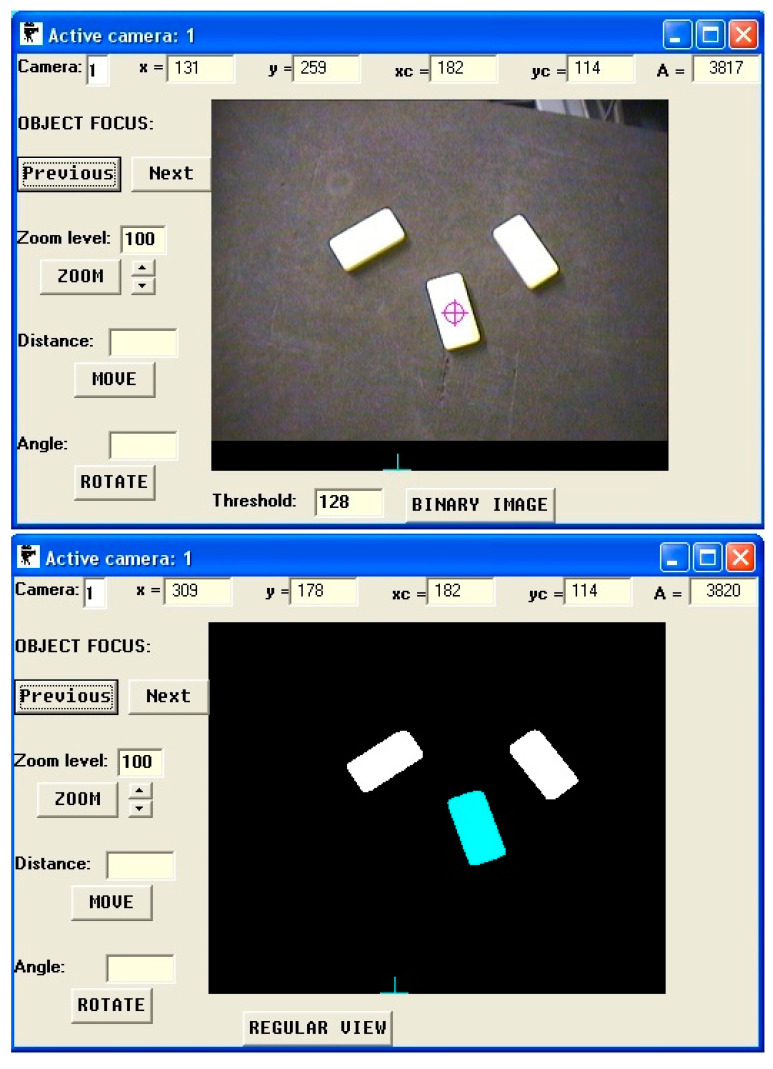
Graphical User Interface of image recognition system used in experiments.

As the use of speech-based communication in medical robotics is still in its infancy stage, the potential of parallel voice control of the robot and of the image processing system has not been studied in practice yet; hence, it is difficult to have full confidence in which functionalities of the image processing system would be willingly used by surgeons in practice (and to what extent). Therefore, for the purpose of the research presented in this paper, a simple custom image recognition system (previously developed in our laboratory) was used in order to find out how effective the speech-based operation of its Graphical User Interface (GUI) was. The GUI of this system is presented in Figure 1.

As far as the speech-based control system is concerned, its core was created using Microsoft Speech SDK. The main aim of the experiment was to practically evaluate the usefulness of voice control based on the skill types discussed in this paper.

This evaluation also comprised a comparison between manual and speech-based control of various functions of the robot, as well as those of the image recognition system. Therefore, a software interface was created to remotely activate the functions of both those system components (e.g., various types of robot motion, operations on the image provided by the camera). The speech-based control system was connected to that interface.

In order to run the various types of scenarios described in this paper, a set of appropriate skills, as well as a set of corresponding voice commands, was defined using RSD and VCD formats. The skills of the robot were as follows:

- Linear motion in a given direction (left, right, up, down, forwards, back) with a given distance;
- Rotation of the end-effector in a given direction (left, right) by a given angle;
- Continuous linear motion in a given direction;
- Continuous rotation of the end-effector in a given direction.

The skills of the image recognition system were as follows:

- Focusing on an object recognized on the screen;
- Image zoom at a given level;
- Image translation with a given distance;
- Image rotation by a given angle.

Functioning of the speech-based control of the robot is presented in Videos S1 and S2 (VideoS1.mp4 and VideoS2.mp4 files available as Supplementary Data). In order to compare the effectiveness of actions controlled manually and by voice, two parameters were measured for each skill:

- Latency between skill launching and start of the appropriate action (in seconds);
- Positioning repeatability (in millimeters or degrees—depending on motion type).

Skill launching is meant as instigation of an action leading to skill execution. For example, it takes place when the operator presses the button on the robot control panel, starts to utter the voice command, or starts to type in data in the appropriate input field of GUI. Positioning repeatability was measured as the greatest difference between positions achieved by the robot for a particular repeatedly executed skill.

Speech-based control of linear robot motion with a given distance or rotation by a given angle consists of uttering an appropriate command containing information about that distance or angle (e.g., “Rotate left by twenty degrees”).

The manual control of such motion is not possible from the robot control panel; therefore, it is performed using GUI. The operator has to use the keyboard to enter the numerical value in an appropriate input field.

Speech-based control of continuous linear or angular motion is performed as a skill of the type “continuous”. The main phrase (e.g., “move right”) is uttered in order to start the motion. When the robot end-effector is approaching the destination point, the operator may decrease the speed (uttering the command “slow down” or “attention”) in order to provide more accurate positioning. The robot ceases to move when the command “stop” or “terminate motion” is uttered.

The manual control of such motions simply consists of pressing and releasing appropriate buttons on the robot control panel. The results of employing both methods for robot skills are summarized in Table 1.

Three values are presented in each cell. They denote, consecutively: maximum value, mean value, and standard deviation. However, the maximum values are of substantial importance because medical applications have to provide full safety to the patient, whereas each overstep (even if its probability is very low) may be a source of hazard.

Although this paper deals mainly with voice-to-action interface, and detailed technical solutions regarding safety are not in its scope, some attention should nevertheless be given to this issue because it has a crucial impact on the possibility of introducing speech-based communication in practice. In scenarios employing continuous action skills, it is assumed by definition that a command terminating the motion must be uttered in the appropriate moment. However, the question arises: what happens when this command—due to some unexpected reason—is not spoken or is wrongly recognized by the speech recognition system? This may lead to a dangerous situation. One possible solution is to impose some restrictions on the length of robot displacement. However, it would probably be difficult to determine one particular value appropriate for all situations. Therefore, the best solution seems to be to provide an emergency stop. Probably the simplest way to arrange it is to customize a foot pedal (which is commonly used with medical robots, e.g., HIWIN) because it will not engage the surgeon’s hands.

As for Table 1, let us briefly discuss its contents now. The measurement of positioning repeatability for motions with a given distance/angle is not applicable because this parameter depends solely on robot positioning repeatability. Whether we apply manual or speech-based control, the results will be exactly the same. In contrast, positioning repeatability in continuous motion depends on more factors: not only robot positioning repeatability, but also the operator’s percipience and reflex (this influences the moment when the button is released or the command “stop” is uttered), as well as skill execution latency, which is different for both control methods. For manual control, this latency is very small, whereas the voice control requires an additional amount of time for command analysis. However, as can be seen from Table 1, differences in positioning repeatability for both control methods are moderate. Repeatability values shown in the table are generally poor from the point of view of medical applications. However, they are mainly influenced by the quality and condition of the robot itself; hence, they are not meaningful in this research. If a better robot were used, these values would be significantly lower. In this research, the meaningful factor is the difference between results achieved for both methods of robot control.

As far as latency between skill launching and motion execution is concerned, there is a significant difference between both methods for continuous motions (the latency for voice control is 5–6 times longer), but the situation is quite different for motions with a given distance/ given angle. This results mainly from the fact that manual control requires the user to type in numerical values in an appropriate input field of the GUI, and sometimes it may take longer than uttering the voice command.

As far as image recognition system skills are concerned, their manual execution is always performed using the GUI, whereas the speech-based control involves uttering commands that must contain all information needed to unambiguously perform the appropriate action.

The results of employing both control methods for the skills of the image recognition system are summarized in Table 2.

As the parameters of actions performed by the image recognition system are unambiguously determined, and the actions themselves are always performed exactly, positioning repeatability is not applicable here. The only indicator of effectiveness remains the latency. The values of latency shown in Table 2 are, certainly, not fully objective, because they depend on a particular user and his/her abilities. For example, the experiments presented in this section were conducted by an operator possessing very good typing skills (using computers in everyday professional practice). Therefore, we may expect that for some other users, the comparison of effectiveness for both control methods could be more in favor of speech-based control.

Manual focus on the object was performed simply by moving the mouse cursor to the appropriate image point. In contrast, speech-based focus on the object was more complex: it was activated by the voice command “Focus on object” followed by automatic recognition of the objects visible on the screen. This action was performed within a multiple choice scenario: switching between objects was performed in response to the voice command “the next one” or “the previous one”. As can be seen from Table 2, the latency is definitely greater for speech-based control.

Image zoom at a given level was performed manually by entering the level value in an appropriate input field. Speech-based zoom was executed in response to an appropriate voice command specifying the zoom level (e.g., “Enlarge the image by fifteen percent”). As can be seen from Table 2, the difference in latencies measured for both methods is not very significant. The same applies to actions consisting in image translation and rotation. Which method offers shorter latency depends strongly on the particular values of parameters that had to be either typed in the input field or specified verbally.

An important benchmark of speech-based interface performance is comparison of voice command recognition rates for various commands. It may lead to conclusions regarding the purposefulness of voice control in various situations, as well as indications regarding the voice command language. Two experiments were conducted. The aim of the first one was to evaluate the recognition rate for all individual commands for the robot (as described in Supplementary Research Data S1). It was assumed that the operator uttered all commands correctly, i.e., only the words from the language vocabulary were spoken. The results are presented in Table 3. The left column contains the names of commands and not the uttered expressions (generally, each command may be expressed in various ways—see Supplementary Data S1).

As can be seen from Table 3, all simple commands (consisting of one word or of a small number of words with constant syntax) were recognized 100% correctly. The recognition rates were lower for commands containing numbers. When the incorrect recognitions were analyzed in detail, it happened that almost all errors resulted from confusion between the words “eight” and “eighty” or “seventy” and “seventeen”, whereby occurrence of the error strongly depended on the next word in the sentence (for example, confusion between “eight” and “eighty” occurred when the phrase “eight degrees” was spoken, but it did not occur for the phrase “eight millimeters”. Two conclusions can be drawn from this experiment. Firstly, further improvement of speech recognition engines could help us to get rid of such problems. Secondly, even contemporary speech recognition engines could bring satisfactory results when appropriate selection of voice command syntax is performed.

However, we cannot always assume that the operator utters only the commands contained in the defined sublanguage. Due to mistakes and effects of so-called spontaneous speech, he may sometimes accidentally utter expressions that are similar to voice commands which he, in fact, does not want to say. The same refers to background speech (e.g., conversation with another person). In order to find out how big the risk is of wrong recognition in such situation, the second experiment was conducted. The operator conversed with another person, and unwanted recognitions were registered. In this way, the commands which were prone to accidental incorrect recognition were determined. The results are summarized in Table 4.

The “background conversation” lasted until 50 accidental recognitions were registered. As can be seen, 50% of those accidental recognitions referred to short expressions “stop” and “faster”. The more complicated the command, the lower the probability of its incorrect recognition. Surprisingly, there was even one case of quite complex expression recognized, namely, “move nine millimeters forwards”. This shows that the use of speech-based interfaces is not desirable in the presence of noise, particularly in medical applications where it may lead to some hazards. It also indicates the importance of various security measures, such as emergency stops, etc.

## 4. Discussion

Two aims were achieved in the research presented in this paper. The first one was to develop and to test a method facilitating creation of voice-controlled medical robotic systems, taking into account possible scenarios of man-machine collaboration in such systems. Robot skill description (RSD) format was proposed in order to facilitate programming of voice control applications. This format, together with the previously developed VCD format for description of voice command language, makes it possible for non-expert users to easily create application-specific scenarios involving robot skills activated by voice commands, whereas the system core needs to be programmed only once by an expert.

In order to test how the speech-based interface would function in practice, a sample voice-controlled system was developed. The experiments described in the previous section helped to achieve another goal: analysis of purposefulness regarding speech-based control of various actions that can be performed within a medical robotic system. This will be discussed now.

As can be seen from Table 1 in the previous section, application of voice control negatively influences the positioning repeatability for robot motions performed within scenarios based on continuous dialog, but this influence seems to be rather moderate. On the other hand, the latency values are several times greater for speech-based control. Nevertheless, when we realize that in the worst case, the overall latency did not exceed 3 s (measured from the beginning of voice command utterance), it may be concluded that voice control of continuous motions may be conditionally applied in medical applications, particularly when we take into account its advantage of freeing the surgeon’s hands. As can be seen in Video S1 in Supplementary Data, controlling this robot skill looks quite reasonable in practice.

In contrast, there are no doubts about the purposefulness of speech-based control of motions with pre-determined distances. As can be seen from Table 1, differences in latency for both control methods are rather small. In this situation, the user friendliness of voice control definitely prevails.

As can be seen from Table 2, similar conclusions can be drawn with respect to actions performed by the image recognition system, although there are some exceptions. Focusing on objects definitely results in greater latency when it is executed using voice commands. Additionally, it is less user friendly because it does not allow the user to choose the object of interest at once when there are more of them on the screen. Nevertheless, even this function could also be controlled by speech in some situations when freeing the surgeon’s hands is the crucial factor.

In summary, the purposefulness of speech-based control of actions executed by medical robotic systems strongly depends on the particular situation and on the operator’s skills and abilities. For example, as mentioned in the previous section, it cannot be determined in advance which control method offers shorter latency because it depends—among others—on particular values of parameters that have to be either typed in the input field or specified verbally. Therefore, we may draw a conclusion that the best solution is to make the voice control system available for the user, parallel to other means of communication. The user will decide which control method is more suitable at the moment.

## 5. Conclusions

Two aims discussed in the previous section were achieved. Firstly, a powerful tool was created that makes it possible to develop speech-based interfaces for medical robots with much higher efficiency than before. The comparison presented at the end of Section 2 does not allow any doubts in this matter. Instead of time-consuming programming in a general-purpose language, it is possible to develop a speech-based interface simply and transparently by creating appropriate skill descriptions in RSD and voice command language descriptions in VCD. The second aim was analysis of the purposefulness of speech-based control of various actions in a medical robotic system. It shows that voice communication with medical robotic systems is efficient; however, further research has to be conducted in order to provide higher reliability of command recognition. One of the possible solutions is careful selection of voice command syntax based on tests performed before the final implementation. The promising results of the research presented in this paper allow us to draw the conclusion that efforts aimed at improvement of speech-based interfaces for medical applications are fully justified and desirable. The research presented in this paper is just a step on the way.

## Figures and Tables

**Table 1:** Effectiveness of robot actions controlled manually and by speech.

Action	Latency [s]		Positioning Repeatability [mm]/[°]	
	Manual	Speech	Manual	Speech
Linear motion with given distance	6.5/5.75/0.75	4.5/4.35/0.23	not applicable	not applicable
Rotation by given angle	3.5/3.25/0.34	5.0/4.75/0.34	not applicable	not applicable
Continuous linear motion	0.5/0.45/0.07	2.5/2.35/0.23	2.0/1.35/0.55	3.5/2.75/0.81
Continuous angular motion	0.5/0.45/0.08	3.0/2.85/0.23	4.5/3.6/0.92	5.0/4.5/0.59

**Table 2:** Effectiveness of vision system actions controlled manually and by speech.

Action	Latency [s]	
	Manual	Speech
Focus on object	0.5	3.0
Image zoom at given level	3.0	4.5
Image translation with given distance	6.0	5.0
Image rotation by given angle	3.0	5.0

**Table 3:** Recognition rates for individual voice commands.

Command	Recognition Rate [%]
MOVE RIGHT	100
MOVE LEFT	100
MOVE UP	100
MOVE DOWN	100
MOVE FORWARDS	100
MOVE BACK	100
ROTATE LEFT	100
ROTATE RIGHT	100
SLOW DOWN	100
SPEED UP	100
MOVE DISTANCE RIGHT	98
MOVE DISTANCE LEFT	97
MOVE DISTANCE UP	99
MOVE DISTANCE DOWN	98
MOVE DISTANCE FORWARDS	97
MOVE DISTANCE BACK	99
ROTATE ANGLE RIGHT	98
ROTATE ANGLE LEFT	96
STOP	100

**Table 4:** Incorrectly recognized expressions sorted by occurrence rate.

Occurrence Rate [%]	Incorrectly Recognized Expression
30	STOP
20	FASTER
16	ATTENTION
12	MOVE DOWN
6	SPEED UP
6	MOVE UP
6	MOVE BACK
2	MOVE RIGHT
2	MOVE NINE MILLIMETERS FORWARDS

## Data Availability

Not applicable.

## Supplementary Materials

The following supporting information can be downloaded at https://www.mdpi.com/article/10.3390/s22239520/s1, Research Data S1: research data S1.doc; Video S1: VideoS1.mp4; Video S2: VideoS2.mp4.
