# Supplementary material for: Scenario-Based Programming of Voice-Controlled Medical Robotic Systems
Source: Sensors (Basel). 2022 Dec 6;22(23):9520. doi: 10.3390/s22239520 (PMC9738457; doi:10.3390/s22239520)
Supplement: Supplementary file 1 [file sensors-22-09520-s001.zip › sensors-2031470-supplementary/research data S1.pdf]

Sublanguage of voice commands in VCD format (file Commands.vcd):

```
#use numbers.vcd

#com terminate
terminate program

#com move distance right
move *number1-999(numbers) *lengthunits to the right : p2*p3

#com move distance left
move *number1-999(numbers) *lengthunits to the left : p2*p3

#com move distance up
move *number1-999(numbers) *lengthunits up : p2*p3

#com move distance down
move *number1-999(numbers) *lengthunits down : p2*p3

#com move distance forwards
move *number1-999(numbers) *lengthunits forwards : p2*p3

#com move distance back
move *number1-999(numbers) *lengthunits back : p2*p3

#def lengthunits
millimeters:1
centimeters:10

#com rotate angle right
rotate *number1-999(numbers) *angleunits to the right

#com rotate angle left
rotate *number1-999(numbers) *angleunits to the left

#def angleunits
degrees: 1
radians: pi()/180

#com move right
move right

#com move left
move left

#com move up
move up

#com move down
move down
```

#com move forwards
move forwards

#com move back
move back

#com rotate right
rotate right

#com rotate left
rotate left

#com slow down
slow down
attention

#com speed up
speed up
faster

#com rotation slow down
slow down
attention

#com rotation speed up
speed up
faster

#com stop
stop
terminate motion

<u>Library of auxiliary phrases in VCD format (file Numbers.vcd):</u>

#def number1-999
*number1-99
*hundreds

#def number1-99
*number1-9
ten :10
*teens
*tens
*tens *number1-9

#def number1-9
one :1
two :2
three :3

four :4
five :5
six :6
seven :7
eight :8
nine :9

#def teens
eleven :11
twelve :12
thirteen :13
fourteen :14
fifteen :15
sixteen :16
seventeen :17
eighteen :18
nineteen :19

#def tens
twenty :20
thirty :30
forty :40
fifty :50
sixty :60
seventy :70
eighty :80
ninety :90

#def full_hundreds
*number1-9 hundred :p1*100

#def hundreds
*full_hundreds
*full_hundreds *number1-99

<u>Skill description in RSD format (file Skills.rsd):</u>

#skill move distance right
type: simple
execute: move right (p1)
confirm: speaker (end of motion)

#skill move distance left
type: simple
execute: move left (p1)
confirm: speaker (end of motion)

#skill move distance up
type: simple
execute: move up (p1)

confirm: speaker (end of motion)

#skill move distance down
type: simple
execute: move down (p1)
confirm: speaker (end of motion)

#skill move distance forwards
type: simple
execute: move forwards (p1)
confirm: speaker (end of motion)

#skill move distance back
type: simple
execute: move back (p1)
confirm: speaker (end of motion)

#skill rotate angle right
type: simple
execute: rotate right (p1)
confirm: speaker (end of rotation)

#skill rotate angle left
type: simple
execute: rotate left (p1)
confirm: speaker (end of rotation)

#skill move right
type: continuous
execute: step right
modify: slow down, speed up
terminate: stop

#skill move left
type: continuous
execute: step left
modify: slow down, speed up
terminate: stop

#skill move up
type: continuous
execute: step up
modify: slow down, speed up
terminate: stop

#skill move down
type: continuous
execute: step down
modify: slow down, speed up
terminate: stop

#skill move forwards
type: continuous
execute: step forwards
modify: slow down, speed up
terminate: stop

#skill move back
type: continuous
execute: step back
modify: slow down, speed up
terminate: stop

#skill rotate right
type: continuous
execute: rotate step right
modify: rotation slow down, rotation speed up
terminate: stop

#skill rotate left
type: continuous
execute: rotate step left
modify: rotation slow down, rotation speed up
terminate: stop

#skill slow down
type: auxiliary
execute: decrease speed

#skill speed up
type: auxiliary
execute: increase speed

#skill rotation slow down
type: auxiliary
execute: decrease rotation speed

#skill rotation speed up
type: auxiliary
execute increase rotation speed

#skill stop
type: auxiliary
execute: stop motion

<u>Robot control procedures (file Execute.prg):</u>

procedure initialize
linear step:=10
rotational step:=30

```
endproc

procedure move right
parameters length
load(dw_[length],0,0)
execute
endproc

procedure move left
parameters length
load(dw_[-length],0,0)
execute
endproc

procedure move up
parameters length
load(dw_0,0,[length])
execute
endproc

procedure move down
parameters length
load(dw_0,0,[-length])
execute
endproc

procedure move forwards
parameters length
load(dw_0,[length],0)
execute
endproc

procedure move back
parameters length
load(dw_0,[-length],0)
execute
endproc

procedure rotate right
parameters angle
position(1,0,0,0,0,[-angle])
load(he_2)
load(ma_1,2)
execute
endproc

procedure rotate left
parameters angle
position(1,0,0,0,0,[angle])
load(he_2)
```

```
load(ma_1,2)
execute
endproc

procedure step right
load(dw_[linear step],0,0)
execute
endproc

procedure step left
load(dw_[-linear step],0,0)
execute
endproc

procedure step up
load(dw_0,[linear step],0)
execute
endproc

procedure step down
load(dw_0,[-linear step],0)
execute
endproc

procedure step forwards
load(dw_0,0,[linear step])
execute
endproc

procedure step back
load(dw_0,0,[-linear step])
execute
endproc

procedure rotate step right
position(1,0,0,0,0,[-rotational step])
load(he_2)
load(ma_1,2)
execute
endproc

procedure rotate step left
position(1,0,0,0,0,[rotational step])
load(he_2)
load(ma_1,2)
execute
endproc

procedure decrease speed
linear step:=linear step div 10
```

```
if linear step<1
 linear step:=1
endif
endproc

procedure increase speed
linear step:=linear step * 10
if linear step>50
 linear step:=50
endif
endproc

procedure decrease rotation speed
rotational step:=rotational step div 15
if rotational step<2
 rotational step:=2
endif
endproc

procedure increase rotation speed
rotational step:=rotational step * 15
if rotational step>30
 rotational step:=30
endif
endproc

procedure stop motion
call initialize
endproc
```
